# Supplementary material for: Random-sequence genetic oligomer pools display an innate potential for ligation and recombination
Source: eLife. 2018 Nov 21;7:e43022. doi: 10.7554/eLife.43022 (PMC6289569; doi:10.7554/eLife.43022)
Supplement: Supplementary file 1. [file elife-43022-supp1.docx]

Table S1.

Quantification of N20>p ligation products (Urea-PAGE, stained with SYBRgold).

|  | - MgCl_2_ | | | + MgCl_2_ | | |
| --- | --- | --- | --- | --- | --- | --- |
| RNA band | raw band intensity (AU) | length-weighted intensity | Relative amount (%) | raw band intensity (AU) | length-weighted intensity | Relative amount (%) |
| N_20_ | 5400 | 270 | 90.5 | 4140 | 207 | 86 |
| N_40_ | 1100 | 27.5 | 9.2 | 1240 | 31 | 13 |
| N_60_ | 50 | 0.83 | ~0.3 | 130 | 2.17 | ~0.9 |
