# Supplementary material for: Random-sequence genetic oligomer pools display an innate potential for ligation and recombination
Source: eLife. 2018 Nov 21;7:e43022. doi: 10.7554/eLife.43022 (PMC6289569; doi:10.7554/eLife.43022)
Supplement: Supplementary file 2. [file elife-43022-supp2.docx]

Table S2.

RNA oligonucleotides. EDC = treated with EDC to generate >p from oligonucleotides ending in 2’/3’-monophosphate.

| Name | Source | Sequence (5’ to 3’) | Description |
| --- | --- | --- | --- |
| C1_20_-N_20_ | IDT, (EDC) | GGCGCGAAAUUAAUACGACUNNNNNNNNNNNNNNNNNNNN(2’/3’p; >p; 3’OH) | >p was produced from EDC treatment of 2’/3’p; 3’OH was obtained by dephosphorylation of 2’/3’P |
| C3_20_-N_20_ | IDT, EDC | CGUCAGAAUGCCUACUUGAGNNNNNNNNNNNNNNNNNNNN>p | >p was produced from EDC treatment of 2’/3’p; 3’OH was obtained by dephosphorylation of 2’/3’P |
| N_20_-C2_20_ | IDT | (5’OH)NNNNNNNNNNNNNNNNNNNNCACGCUGCCCGUAUGUAUAU |  |
| N_20_-C4_20_ | IDT | (5’OH; 5’p)NNNNNNNNNNNNNNNNNNNNGAGUCCCUUGCCAUGCAACG |  |
| N_20_>P | IDT, EDC | NNNNNNNNNNNNNNNNNNNN>p | >p was produced from EDC treatment of 2’/3’p; |
| FITC-N_20_>p | IDT, EDC | /56FAM/-NNNNNNNNNNNNNNNNNNNN>p | >p was produced from EDC treatment of 2’/3’p; |
| N_20_A_10_ | IDT | NNNNNNNNNNNNNNNNNNNNAAAAAAAAAA |  |
| FITC-N_20_ | IDT | NNNNNNNNNNNNNNNNNNNN |  |
| 5H4 | IDT,EDC | CGUCAGAAUGCCUACUUGAGUUGAGUGACUAGUCGGGUUG>p | >p was produced from EDC treatment of 2’/3’p; |
| 3HF | IDT | AUGACCAGGGCGUUGGGGAACACGCUGCCCGU/36FAM/ |  |
| 5H4T1 | IDT, EDC | CCAUCCUCUAGUUG>p | >p was produced from EDC treatment of 2’/3’p; |
| 3H4T1 | IDT | AUACCCACACCACA/36FAM/ |  |
| H4_splintG | IDT | GUGGUGUGGGUAUCAACUAGAGGAUGG |  |
| H4_splintA | IDT | GUGGUGUGGGUAGAAUGCUAGAGGAUGG |  |
| H4_min-P | IDT, EDC | CGUCAGAAUGCCUGUACGCGGGUUG>p | >p was produced from EDC treatment of 2’/3’p; |
| H4_min-F | IDT | AUGACGACAC/36FAM/ |  |
| J4_min-P | IDT, EDC | CGUCCGAAAUUCCGUACGCGGAACG>p | >p was produced from EDC treatment of 2’/3’p; |
| J4_min-F | IDT | GAGGACGACAC/36FAM/ |  |
| 5-Rseq1>p | IDT, EDC | CGUCAGAAUGCCUACUUGAGCGGUGGAGGCGGCAGAAGUC>p | >p was produced from EDC treatment of 2’/3’p; |
| 3-Rseq1-FAM | IDT | GACGCUGGCCGGCGCAUGGUCACGCUGCCCGUAUGUAUAU/36-FAM/ |  |
| 5-Rseq2>p | IDT, EDC | GGCGCGAAAUUAAUACGACUGGUGGUAACGUGCUAGUGGU>p | >p was produced from EDC treatment of 2’/3’p; |
| 5-Rseq2_shrt>p | IDT, EDC | GGCGCGGAAACGUGCUAGUGGU>p | >p was produced from EDC treatment of 2’/3’p; |
| 3-Rseq2-FAM | IDT | GAGGCGACUCGUGACAAAAAGAGUCCCUUGCCAUGCAACG/3-FAM/ |  |
| 3-Rseq2_shrt-FAM | IDT | GAGGCGAAAGCCUUGCCAUGCAACG/36-FAM/ |  |
| 5-Rseq3>p | IDT, EDC | GGCGCGAAAUUAAUACGACUGUCAAGUAUAGCUUGGAGUU | >p was produced from EDC treatment of 2’/3’p; |
| 3-Rseq3-FAM | IDT | GCGGUUAGGAGGAGAUGAUAGAGUCCCUUGCCAUGCAACG/3-FAM/ |  |
| 5-Rseq4>p | IDT, EDC | CGUCAGAAUGCCUACUUGAGGGGCGCAGGAGAUGACGUGG>p | >p was produced from EDC treatment of 2’/3’p; |
| 3-Rseq4-FAM | IDT | ACGUCACACGGUUGGGUUGUCACGCUGCCCGUAUGUAUAU/36-FAM/ |  |
