# Supplementary material for: Random-sequence genetic oligomer pools display an innate potential for ligation and recombination
Source: eLife. 2018 Nov 21;7:e43022. doi: 10.7554/eLife.43022 (PMC6289569; doi:10.7554/eLife.43022)
Supplement: Supplementary file 3. [file elife-43022-supp3.docx]

Table S3.

DNA oligonucleotides.

| Name | Source | Sequence (5’ to 3’) | Description |
| --- | --- | --- | --- |
| C1_fw | IDT | GGCGCGAAATTAATACGACT | RT-primer for srRNA |
| C2_rv | IDT | ATATACATACGGGCAGCGTG | RT-primer for srRNA |
| C3_fw | IDT | CGTCAGAATGCCTACTTGAG | RT-primer for srRNA |
| C4_rv | IDT | CGTTGCATGGCAAGGGACTC | RT-primer for srRNA |
| Appa-3RND-Cy5 | IDT as 5’ P,  5’adenylation by enzymatic reaction | /App/GCAAGTGAACTTCTGTCCAG/3Cy5Sp/ | Preadenylated linker for RT-recovery of naive N20>p ligation products and N20-preligation material |
| 3_RND_rev | IDT | CTGGACAGAAGTTCACTTGC | Reverse cDNA amplification primer for N20>p ligation products and N20-preligation material |
| 5_RNDM linker | IDT | /56FAM/AGTTACTATGGCAGCCGAGA | Adapter oligo for 1^st^ strand synthesis cDNA of N_20_>p ligation products and N_20_-preligation material |
| 5_RND_fw | IDT | AGTTACTATGGCAGCCGAGA | Forward cDNA amplification primer for N_20_>p ligation products and N_20_-preligation material. |
| miRNA Cloning Linker 1 | IDT | /5rApp/CTGTAGGCACCATCAAT/3ddC/ | Adapter oligo for srRNA bait pre-ligation sequencing |
| miRNA_CL1_rev | IDT | ATTGATGGTGCCTACAG | Reverse primer for RT using miRNA Cloning Linker 1 |
| P5_5RND | IDT | AATGATACGGCGACCACCGAGATCTACACTCTTTCCCTACACGACGCTCTTCCGATCTNNN[BC6]AGTTACTATGGCAGCCGAGA | Forward primer for library preparation from N_20_>p ligation product cDNA; [BC6] = 6 nt NEXTflex barcode |
| P3_3RND | IDT | CAAGCAGAAGACGGCATACGAGATCGGTCTCGGCATTCCTGCTGAACCGCTCTTCCGATCTCTGGACAGAAGTTCACTTGC | Reverse primer for library preparation from cDNA of adapter ligated N_20_>p ligation products / N20 pre-ligation RNA. |
| P5_C1 | IDT | AATGATACGGCGACCACCGAGATCTACACTCTTTCCCTACACGACGCTCTTCCGATCTNNN[BC6]GGCGCGAAATTAATACGACT | Forward primer for library preparation from srRNA C1-bait cDNA products; [BC6] = 6 nt NEXTflex barcode |
| P3_C2 | IDT | CAAGCAGAAGACGGCATACGAGATCGGTCTCGGCATTCCTGCTGAACCGCTCTTCCGATCTATATACATACGGGCAGCGTG | Reverse primer for library preparation from srRNA prey-C2 cDNA products. |
| P5_C3 | IDT | AATGATACGGCGACCACCGAGATCTACACTCTTTCCCTACACGACGCTCTTCCGATCTNNN[BC6]CGTCAGAATGCCTACTTGAG | Forward primer for library preparation from srRNA / srXNA C1.2-bait cDNA product; [BC6] = 6 nt NEXTflex barcode |
| P3_C4 | IDT | CAAGCAGAAGACGGCATACGAGATCGGTCTCGGCATTCCTGCTGAACCGCTCTTCCGATCTCGTTGCATGGCAAGGGACTC | Reverse primer for library preparation from srRNA / srXNA prey-C2.2 cDNA product. |
| P5_C2 | IDT | AATGATACGGCGACCACCGAGATCTACACTCTTTCCCTACACGACGCTCTTCCGATCTNNN[BC6]ATATACATACGGGCAGCGTG | Primer for library generation for the reverse complement library of the prey-C2 pre-ligation srRNA. |
| P5_C4 | IDT | AATGATACGGCGACCACCGAGATCTACACTCTTTCCCTACACGACGCTCTTCCGATCTNNN[BC6]CGTTGCATGGCAAGGGACTC | Primer for library generation for the reverse complement library of the prey-C2.2 pre-ligation srRNA. |
| P3_miRNA_CL1_rv | IDT | CAAGCAGAAGACGGCATACGAGATCGGTCTCGGCATTCCTGCTGAACCGCTCTTCCGATCTATTGATGGTGCCTACAG | Reverse primer for library preparation from C1- / C1.2- bait or prey-C2 / -C2.2 pre-ligation srRNA. |
| FD | IDT | CCCTTATTAGCGTTTGCCA | forward primer for srXNA synthesis |
| C1_N20_temp | IDT | NNNNNNNNNNNNNNNNNNNNAGTCGTATTAATTTCGCGCCTGGCAAACGCTAATAAGGG | template for XNA C1-bait |
| C2_N20_temp | IDT | ATATACATACGGGCAGCGTGNNNNNNNNNNNNNNNNNNNNTGGCAAACGCTAATAAGGG | template for XNA prey-C2 |
| GB2_3soupRTmix | IDT | CGGCCATCACTACAACCACmAmTmAmTmAmCmAmTmAmCmGmGmGmCAGCGTG | RT primer for srHNA and AtNA; m = 2’OMe |
| GB2_3soupRT | IDT | CGGCCATCACTACAACCACATATACATACGGGCAGCGTG | RT primer for srANA |
| GB2 | IDT | CGGCCATCACTACAACCAC | Forward primer for 1^st^ cDNA PCR of srXNA products; reverse primer: C1_fw |
| Tag3_polyT | IDT | CAAGGTAGTGCTGTTCGTTTTTTTTTTTTTTTTTTTT | SMARTer RT primer for N20A10 RNA (P1 band) |
| Tag3_A20N1 | IDT | CAAGGTAGTGCTGTTCGAAAAAAAAAAAAAAAAAAAAN | SMARTer RT primer for polyuridinylated N20A10 RNA (P1 & P2 band) |
| Tag3 |  | CAAGGTAGTGCTGTTCG | PCR amplification primer |
| Smarter5PCRPIIA | IDT | AAGCAGTGGTATCAACGCAGAGT | General PCR amplification primer for SMARTer cDNA |
| E5112_H4 | IDT | GCCCTGGTCAGTCAGCTGACTCGAAAACCCGAC | DNAzyme for 3’-5’ linked full-length H4 RNA |
| E1111_J4 | IDT | GTGTCGTCCTTGTCAGCGACACGAACGTTCCGCGTACGGAATTTCGGACG | DNAzyme for 3’-5’ linked full-length J4 RNA |
| P5_tag3_bar | IDT | AATGATACGGCGACCACCGAGATCTACACTCTTTCCCTACACGACGCTCTTCCGATCTNNN[BC6]CAAGGTAGTGCTGTTCG | Forward primer for library generation of SMARTer cDNA (sequencing of reverse complement products); BC6] = 6 nt NEXTflex barcode |
| P3_Smarter5PCRPIIA | IDT | CAAGCAGAAGACGGCATACGAGATCGGTCTCGGCATTCCTGCTGAACCGCTCTTCCGATCTAAGCAGTGGTATCAACGCAGAGT | Reverse primer for library generation of SMARTer cDNA (sequencing of reverse complement products) |
| J4-min splint | IDT | GTGTCGTCCTCCGTTCCGCGTAC | Splint to create 3’-5’ ligated J4-min with T4-RNA Ligase 2 |
